# Setting matters: Associations of nurses’ attitudes towards people with dementia

**DOI:** 10.1002/nop2.198

**Published:** 2018-10-12

**Authors:** Regula Blaser, Jeanne Berset

**Affiliations:** ^1^ Institute on Ageing University of Applied Sciences Bern Switzerland

**Keywords:** dementia, nurses, nursing, older people, Switzerland

## Abstract

**Aim:**

Identify associations between attitudes of nurses towards people with dementia and their socio‐demographic (age and gender) and work‐related characteristics (employment, work experience, nursing degree, care setting).

**Design:**

The study was designed in a cross‐sectional way, collecting self‐reported questionnaire data.

**Methods:**

Nurses (*N* = 417) completed the 20‐item Dementia Attitude Scale questionnaire, including socio‐demographic and work characteristics. Data were analysed using descriptive statistics, group mean comparison, correlation and regression analysis.

**Results:**

The attitudes of nurses towards people with dementia were only related to the care setting. Attitudes of nurses working in dementia‐specialized long‐term care institutions were significantly more positive compared with those working in mixed long‐term care institutions and in the homecare setting. The other variables, such as age, gender, employment, nursing degree and work experience, were not associated with the attitudes.

## 1 INTRODUCTION

Currently, about 119,000 persons in Switzerland suffer from some form of dementia (Schweizerische Alzheimervereinigung, 2014a); 50% of people diagnosed with dementia are still living at home (Schweizerische Alzheimervereinigung, 2014b), and half of them are cared by professional home care services (for instance “Spitex”; Ecoplan, 2013). Home care nurses care for people living at home with various degrees physical and/or mental disabilities. Most of them are mildly to moderately limited in their activities of daily living (Wächter et al., 2015). The situation of a person with dementia in an early or middle stage is very demanding for home care nurses, as affected persons are often aware of their increasing losses in the course of the expected progression of the disease and react with diverse symptoms, such as grief, depression, anxiousness or aggression (Schweizerische Alzheimervereinigung, 2014c).

The other half of the persons diagnosed with dementia are living in nursing homes. In contrast to the home care setting, persons living in institutions of long‐term care tend to be more limited in their activities of daily living. In the German part of Switzerland, there are nearly 1,300 institutions of long‐term care. Either they are integrated facilities, which care, among others, for people with dementia, or they are special care units, which are exclusively for people with dementia. The challenge of integrated facilities is that nurses must cope with the needs of people with dementia and with the needs of people with other diagnoses, while in specialized long‐term care, nurses can focus exclusively on the needs of people with dementia. In integrated facilities, 64.5% of the residents are diagnosed or suspected to suffer from dementia (Schweizerische Alzheimervereinigung, 2014b).

## 2 BACKGROUND

Previous studies already indicated that well‐being and quality of life of the persons suffering from dementia depend on the nursing staff involved (Kada, Nygaard, Mukesh, & Geitung, 2009). Quality of nursing is known to be highly related to the knowledge about and the attitudes towards dementia held by professionals (Norbergh, Helin, Dahl, Hellzén, & Asplund, 2006). This connection has already been intensely studied by social psychologists and has been considered as essential for the quality of nursing (Åström, Nilsson, Norberg, Sandman, & Winblad, 1991; Fessey, 2007; Stahlberg & Frey, 1992, Soderhamn, Lindencrona, & Gustavsson, 2001).

Various studies showed that nursing home staff tends to have a more negative than positive perception of dementia patients (Asplund & Norberg, 1993; Brodaty, Draper, & Low, 2003). Interestingly, however, the contradictory effects were also found (Kada et al., 2009; Moyle, Murfield, Griffiths, & Venturato, 2011; Spector & Orrell, 2006). Norbergh et al. (2006) for instance found that nurses had a positive to neutral perception of their dementia patients. In contrast to the studies conducted by Asplund and Norberg (1993) and Brodaty et al. (2003) where nurses had to rate fictitious patients, in the study conducted by Norbergh Helin Dahl Hellzén and Asplund (2006), the nurses had to rate real dementia patients. The reasons for the discrepancy in the results might be that the personal experiences and personal relationships and the upcoming adoption of the personhood approach (Kitwood, 1997, 2004 ) in long‐term care evoked more positive attitudes towards dementia patients.

Researchers have further focused on the factors that influence nurses’ attitudes towards people with dementia. Addressed were factors such as workplace characteristics, socio‐demographic characteristics and dementia knowledge (Åström et al., 1991; Brodaty et al., 2003; Kada et al., 2009¸ Moyle et al., 2011; Zimmerman et al., 2005).

Care staff’s attitudes towards people with dementia are positively correlated with job satisfaction (Moyle et al., 2011; Zimmerman et al., 2005) and negatively with stress and burnout symptoms (Åström et al., 1991; Brodaty et al., 2003; Zimmerman et al., 2005).

Regarding socio‐demographic characteristics, nurses aged 50+ and work experience of less than 10 years (Kada et al., 2009) were associated with a less positive attitude towards dementia. In contrast, Åström et al. (1991) found no differences in attitudes towards people with dementia with regard to the staff’s age or time at present place of work. In the sample of Brodaty et al. (2003), age had no influence on nurses’ attitudes towards dementia either.

Several studies found that Registered Nurses, who have better theoretical dementia knowledge, show more positive attitudes towards residents with dementia than nurses with lower levels of education (Åström et al., 1991; Mellor, Greenhill, & Chew, 2007; Kada et al., 2009). Furthermore, nurses with specialized training in geriatrics, psychiatry or dementia care are reported to have more favourable attitudes than nurses lacking this training (Kada et al., 2009; Zimmerman et al., 2005). Moyle et al. (2011) suggested that such training courses seek to move care staff away from the focus on deficits towards a greater appreciation of the person’s abilities and therefore can positively influence attitudes and behaviour.

## 3 AIM

In sum, nurses’ attitudes towards people with dementia are of great importance. Nurses’ attitudes influence their interactions with persons with dementia and subsequently the quality of the affected persons’ life. Nevertheless, as results are contradictious, more research on the factors influencing these attitudes is needed. To change nurses’ attitudes in a positive direction, knowledge of the influencing factors is needed. Therefore, the aim of this study was to examine the relations between various socio‐demographic and workplace characteristics of nurses and attitudes towards people with dementia.

## 4 THE STUDY

### 4.1 Design

A cross‐sectional research study was conducted involving a survey with a sample of Swiss nurses caring for people with dementia in home care and in long‐term institutional care settings.

### 4.2 Setting

Data were collected in the German‐speaking part of Switzerland, which comprises approximately two‐thirds of the Swiss population or 4.5 million people (https://www.bfs.admin.ch/bfs/de/home/statistiken/bevoelkerung/sprachen-religionen/sprachen.assetdetail.2244919.html#;; accessed 13 August 2018). There are nearly 1,300 institutions of long‐term care in this part of Switzerland. Either they are integrated facilities, which care, among others, for people with dementia, or they are special care units, which are exclusively for people with dementia. In integrated facilities, 64.5% of the residents are diagnosed or suspected to suffer from dementia (Schweizerische Alzheimervereinigung, 2014b).

Among 50% of people with dementia live at home and are cared for by informal or professional caregivers (Schweizerische Alzheimervereinigung, 2014b). The most widely used professional home care service in Switzerland is called Spitex. Spitex is organized locally with approximately 450 organizations in German‐speaking part of Switzerland. The 55% of Spitex clients are diagnosed or suspected to suffer from dementia (Ecoplan, 2013).

Nursing staff in institutional and in the home care setting are put into three categories, according to their academic degree in nursing: nursing aides, licensed nurses, Registered Nurses. According to the staffing schedule of nurses, in institutions of long‐term care there are 40%–50% nursing aides, 24%–30% licensed nurses and 16%–20% Registered Nurses (https://www.vbb-abems.ch/Infos-Downloads/P1kCo/?m=1&c=30F471CD-0C06-36CD-903EB090C40424FE; accessed 13 August 2018). In Spitex organizations, the staffing schedule depends on the service offered.

Data were collected in two waves. The first wave was completed in 2013 with a paper‐and‐pencil version of the questionnaire (*N* = 70). The second wave took place in 2016 and 2017 with an online‐based version of the questionnaire (*N* = 347), which was not available in 2013 yet. Analyses showed no differences in samples’ socio‐demographic variables, work characteristics, or mean score in the Dementia Attitudes Scale (DAS). Therefore, data of the samples were merged for the analyses.

### 4.3 Procedures

Information about the study was presented to the nursing directors and managers of the institutions. The 50 institutions of long‐term care and 18 Spitex organizations agreed to participate in the study. The nursing directors of the respective institutions distributed either the paper version or the link to the online questionnaire to the nursing staff, consisting of Registered Nurses, licensed nurses and nursing aides. Participants could fill in the questionnaire during working time. The sole exclusion criterion was a lack of proficiency in the German language, which would keep participants from understanding and filling in the questionnaire. Each questionnaire was accompanied by an information letter about the study and, if required, a postage‐paid return envelope. Participants were asked to complete the questionnaire in two weeks. After these two weeks, a reminder was sent to the nursing directors to increase response rate. Participants could participate for two more weeks. Participants did not receive monetary compensation for filling in the questionnaire. As we used a snowball sampling, the response rate cannot be determined.

### 4.4 Measurements

#### 4.4.1 Attitudes

To assess nurse’s attitudes towards older people with dementia, data were collected using the German version of the Dementia Attitudes Scale (DAS; Peng, Moor, & Schelling, 2011). The researchers selected this scale because of its empirically affirmed reliability (Cronbach's alpha for the total scale *α* = 0.87) and validity in a Swiss sample (Peng et al., 2011). The original English version was developed by O'Connor and McFadden (2010). The scale consists of 20 positively or negatively formulated items. The factors of the scale are labelled “dementia knowledge” (item example: “People with ADRD can enjoy life.”) and “social comfort” (item example: “I feel confident around people with ADRD.”). Participants are asked to give their degree of agreement on a 7‐point Likert scale, ranging from 1 ‐ 7 (1 = strongly disagree, 7 = strongly agree). Higher scores reflect a more positive attitude. Maximum score is 140 points.

#### 4.4.2 Socio‐demographic and work characteristics

The questionnaire included different socio‐demographic and work characteristics variables. In detail, the continuous variables age, experience with people suffering from dementia and level of employment were integrated. Furthermore, the categorical variables gender (male/female), degree in nursing (nursing aides/practical nurses/licensed nurses) and care setting (special care units for persons with dementia/mixed long‐term care institutions/home care setting) were collected.

### 4.5 Analysis

Data were analysed using the Statistical Package for the Social Sciences (SPSS) version 24. Analyses containing continuous variables were conducted with the row scores. Predefined categories of categorical variables were used for statistical analyses. For each participant, the total‐DAS‐scale sum score was calculated. Descriptive data analyses were used to describe characteristics of the participants. Correlations were used to give a first overview of the relations between the variables and to determine about the independency of variables as precondition for multiple regression analyses. Mann–Whitney tests were used to examine the effect of gender on attitudes towards people with dementia. Furthermore, Kruskal–Wallis tests were used to compare the mean values of DAS scores between different groups of respondents with more than two categories. Dunn–Bonferroni tests were used for pairwise comparisons. Finally, multiple regression analyses were calculated to show in detail how the variables are connected and how much variance of the attitudes of nurses towards older people with dementia is explained by socio‐demographic and work characteristic variables.

### 4.6 Ethics

Nursing directors and managers of the institutions permitted their nurses to take part in this study and to fill in the questionnaire during work time. Nurses were informed that their participation was voluntary, and anonymity was guaranteed. The regional Ethical Committee was informed about the study.

## 5 RESULTS

### 5.1 Characteristics of the participants

The sample consisted of 468 nurses that completed the questionnaire. After exclusion of cases with missing data, a total sample of 417 could be achieved and was used for all subsequent analysis. A total of 376 (90%) study participants were women and 41(10%) were men. Average age of the sample was 43 years (*SD* 13.34). A total of 141 (34%) were nursing aides, 112 (27%) licensed nurses and 164 (39%) Registered Nurses. At the time of the study, 222 (53%) worked in long‐term care institutions specialized in the care for older people with dementia. A total of 134 (32%) nurses were working in mixed long‐term care institutions and 61 (15%) in the home care setting. In sum, participants stem from 50 long‐term care institutions and 18 Spitex organizations located in different cantons in the German‐speaking part of Switzerland. The institutions varied regarding size and location (rural or urban). Participants had an average work experience of 91.3 months (*SD* 81.75) in the care of people with dementia. 257 (61%) of the study participants had a level of employment between 80% and 100% (full‐time) (mean = 76.5, *SD* 20.2). The total sample mean score for the DAS was 114.67 (*SD* 13.51) with a range from 74 to 140 (Table 1).

**Table 1:** Socio‐demographic and work characteristics and DAS score of the sample

Variable	Frequency	Percentage (%)
Gender		
Female	376	90
Male	41	10
Degree in nursing		
Nursing aides	141	34
Licensed nurses	112	27
Registered Nurses	164	39
Type of care setting		
Home care setting	61	15
Mixed long‐term care	134	32
Specialized long‐term care	222	53

	Mean	*SD*
Age (years)	43	13.34
Current level of employment (%)	76.5	20.2
Experience working with people with dementia (months)	91.3	81.75
DAS score	114.67	13.51

Note

*N* = 417.

### 5.2 Correlations of nurses’ attitudes towards older people with dementia

Table 2 shows the correlations of the variables included. Two variables were significantly positively correlated with the DAS score: care setting *V = *0.467 (*p* ≤ 0.001) and work experience with people with dementia *ρ* = 0.113 (*p* ≤ 0.05). The other variables such as gender, age, level of employment and degree in nursing, however, did not significantly correlate with nurses’ attitudes towards people with dementia.

**Table 2:** Correlations among all variables collected

	1	2	3	4	5	6
1 Gender[Link]	–					
2 Age	0.420** [Link]	–				
3 Level of employment	0.303** [Link]	−0.371***, [Link]	–			
4 Work experience	0.049[Link]	0.380***, [Link]	−0.037[Link]	–		
5 Degree in nursing[Link]	0.049[Link]	0.456***, [Link]	0.114[Link]	0.120*, [Link]	–	
6 Care setting[Link]	0.036[Link]	0.407**, [Link]	0.225[Link]	0.512[Link]	0.168***, [Link]	–
7 DAS score	0.362[Link]	0.077	0.035	0.113*	0.347[Link]	0.467***, [Link]

Notes

*N* = 417.

Spearman correlations two‐tailed.

1 = female, 2 = male,

1 = nursing aides, 2 = licensed nurses; 3 = registered nurses

1 = home care setting, 2 = mixed long‐term care setting, 3 = special care units for persons with dementia

Cramer’s V, as gender, degree in nursing and care setting are all nominal variables.

Pearson correlations two tailed

* *p* ≤ .05, ** *p* ≤ .01, ***p ≤ .001.

Mean values of nurses’ attitudes towards people with dementia, depending on the different care settings, are presented in Table 3. The Kruskal–Wallis test shows significant group differences (*χ* ^2^ = 87.5, *df* = 2, *p*<0.001). Dunn–Bonferroni tests were used for pairwise group comparisons. The mean attitudes score of nurses working in specialized long‐term care institutions was significantly more positive than the attitudes scores of nurses working in home care setting (*z* = 7.6, *p*<0.001) or in mixed long‐term care institutions (*z* = 7.4, *p* = 0.000). Although nurses in the mixed long‐term care had a higher DAS score than home care nurses, this difference is not statistically significant (*z* = 1.9, *p* = 0.167).

**Table 3:** Mean values of nurses’ attitudes towards older people with dementia (DAS score) according to different care settings

	Home care setting	Mixed long‐term care setting	Special care units for persons with dementia	Group comparison, significance level
*n*	61	134	222	
Mean	105	109	121	*p* < 0.001[Link] Specialized > home care
Median	104	113	122	
Mode	91	120	125	
*SD*	13.75	13.97	9.89	*p* < 0.001[Link] Specialized > mixed

*N* = 417

*p*‐values for group comparisons based on Dunn‐Bonferroni‐ test.

Further Kruskal–Wallis tests with the independent variables work experience, degree in nursing and age showed no significant group differences in the mean DAS score. The results of the Mann–Whitney tests did not reveal significant group differences in the mean DAS score for the variables gender and level of employment.

### 5.3 Regression analyses

Table 4 shows the results of the regression analyses conducted. Based on the results of the correlation matrix in Table 2, the care setting was entered in the last step of the regression analysis (assuming this variable has the highest predictive power). Control variables such as age, gender, degree in nursing, level of employment and work experience did not explain any considerable portion of the variance in the dependent variable. The care setting allowed for a significant prediction of the attitudes score and explains 21.3% of the variance in the DAS score.

**Table 4:** Regression analysis to predict nurses’ attitudes towards older people with dementia (DAS score)

	*B*	*SE B*	*β*	*R^2^*	*∆R^2^*	VIF
1 Gender[Link]	−0.262	2.03	−0.006	0.006		1.392
Age	0.071	0.052	0.017			
2 Level of employment	−0.024	0.033	−0.036	0.007	0.001	1.254
3 Degree in nursing[Link]	0.737	0.719	0.047	0.009	0.002	1.096
4 Work experience	0.014	0.008	0.087	0.014	0.005	1.233
5 Type of care setting	8.848	0.833	0.477***	0.227	0.213***	1.071

*N* = 417.

Regression analysis, enter‐method with five steps.

1 = female, 2 = male.

1 = nursing aides, 2 = licensed nurses, 3 = registered nurses. *B* = unstandardized regression coefficient, *SE B* = standardized error, *β* = standardized regression coefficient, R^2^ = explained variance, ∆R^2^ = change in R^2^ (additional explained variance),

* *p* ≤ .05, ** *p* ≤ .01, ***p ≤ .001.

## 6 DISCUSSION

The aim of this study was to examine the associations between various socio‐demographic and workplace characteristics and attitudes towards people with dementia in a sample of Swiss nurses with different education levels and of different care settings. 417 nurses participated in the study: 222 (53%) worked in long‐term care institutions specialized in the care for older people with dementia. 134 (32%) nurses were working in mixed long‐term care institutions and 61 (15%) in the home care setting. According to our results, care setting and work experience with people with dementia significantly correlated with nurse’s attitudes towards older people with dementia. Pairwise group comparison test showed that the mean attitude score of nurses working in specialized long‐term care institutions was significantly more positive than the attitude score of nurses working in home care setting or in mixed long‐term care institutions. Furthermore, the mean attitude score of nurses in mixed long‐term care institutions was more positive compared with the mean score of nurses in the home care setting. Regression analysis showed that the care setting allows for a significant prediction of nurses’ attitudes towards people with dementia.

Nurses working in the home care setting must deal with various degrees of physical and/or mental disabilities. People are mostly mildly to moderately limited in their activities of daily living (Wächter & Bommer, 2015). Thus, caring for persons suffering from dementia in an early or middle stage might be especially demanding for those nurses, as affected persons are often aware of their increasing losses and react with diverse symptoms such as grief, depression, anxiousness or aggression (Schweizerische Alzheimervereinigung, 2014c). Additionally, informal caregivers, such as relatives, are suffering from the strains of their caregiving (Ecoplan, 2013; Wilz, Adler, Gunzelmann, & Brähler, 1999; Zank, Schacke, & Leipold, 2007). Therefore, nurses working in the home care setting might get a rather negative view of the situations people with dementia live in. Furthermore, Wächter and Bommer (2015) showed that a high percentage of home care clients are not long‐term clients but people who need timely limited professional support after hospitalization or acute illness. Thereby, home care nurses’ experience in interactions with people suffering from dementia is limited and the so‐called “challenging behaviour” of the affected people might be more difficult to handle for them. Training programmes for home care nurses should cover a wide range of health‐related themes, whereof dementia is probably not the most prominent. Summing up, these points could explain why home care nurses have the lowest DAS score in this study.

In mixed long‐term care settings, half to two‐thirds of the residents are diagnosed with dementia (Schweizerische Alzheimervereinigung, 2014b). Nurses working in this setting must cope with the needs of people with dementia and with the needs of people with other diagnoses. This is a challenge with which nurses in specialized long‐term care are not confronted. This opportunity to focus only on the needs of people with dementia leads to higher job satisfaction in nurses in specialized long‐term care in comparison with nurses in mixed long‐term care (Oppikofer, Lienhard, & Nussbaumer, 2009; Pekkarinen et al., 2006). Dissatisfaction at work has consistently shown to be associated with nurses’ negative attitudes towards people with dementia (Åström et al., 1991; Brodaty et al., 2003; Moyle et al., 2011; Zimmerman et al., 2005). In specialized long‐term care, training programmes are chosen or designed based on the needs nurses have in caring exclusively for people with dementia. This leads to deeper dementia knowledge in nurses in specialized long‐term care (Grant, Kane, Potthoff, & Ryden, 1996; Grant, Potthoff, Ryden, & Kane, 1998; Teresi, Grant, Holmes, & Ory, 1998). Training in mixed long‐term care has to cover a wider range of themes.

There exists an alternative explanation for the results found. According to various conversations the authors had with nurses of specialized long‐term care institutions, the aspects of the setting do not influence nurses’ attitudes towards people with dementia, rather nurses’ attitudes are essential in the choice for a certain care setting. Nurses who decided to work in a specialized long‐term care institution often report to have experienced a high affinity for people with dementia when working with older people. These reports were also found online (https://www.derwesten.de/staedte/sprockhoevel/tagespflege-fuer-an-demenz-erkrankte-id10571454.html?keepUrlContext=true; accessed 13 August 2018). However, empirical literature supporting this hypothesis could not be found.

### 6.1 Strengths and limitations

Similar studies included 49–291 participants. In comparison, the present study comprises a big sample which allowed to conduct all indicated statistical analyses with relatively big subsamples. Data were collected in different, urban and rural regions of German‐speaking part of Switzerland. Institutions and organizations with various amounts of residents or clients were involved. All care settings relevant for people with dementia were represented. However, as the concrete number of the population, that is nurses caring for older people with dementia in German‐speaking part of Switzerland is unknown, we cannot be sure that the sample is representative.

One possible limitation of this study is that self‐assessment questionnaires underlie to social desirability bias. Unfortunately, the nationality of one fourth of the sample could not be assessed and could therefore not be included in the statistical analyses. Among the respondents, only ten percentage were not of Swiss nationality. This proportion seems to be too low to be representative. Merçay, Burla, and Widmer (2016) report a proportion of 15%–19% of immigrated nursing staff in long‐term care.

The paper‐and‐pencil test and the link to the online questionnaire were distributed via snowball. Therefore, the response rate could not be determined.

## 7 CONCLUSION

The presented results indicate that nurses’ attitudes towards people with dementia were associated with the care setting. However, further research is needed to understand the underlying mechanisms. Then, measures for practice intervention should be derived. The aim of such interventions would be to focus on enhancing nurses’ attitudes towards people with dementia in all non‐specialized care settings.

The presented results go line with existing studies showing that further dissemination of the person‐centred care approach in every care setting is a promising way to positively influence nurses’ attitudes. An equally promising way is to give dementia education and training for all nurses working with people with dementia, regardless of their degree in nursing and type of care setting.

## CONFLICTS OF INTEREST

The authors declare no conflict of interest.

## AUTHOR CONTRIBUTIONS

RB, JB: Study design. JB, RB: Data collection and analyses. RB, JB: Manuscript preparation. All authors have agreed to the final version of the manuscript.

- substantial contributions to conception and design, acquisition of data or analysis and interpretation of data;
- drafting the article or revising it critically for important intellectual content.
